# Herpes Simplex Virus 1 Can Bypass Impaired Epidermal Barriers upon *Ex Vivo* Infection of Skin from Atopic Dermatitis Patients

**DOI:** 10.1128/jvi.00864-22

**Published:** 2022-08-15

**Authors:** Maureen Möckel, Nydia C. De La Cruz, Matthias Rübsam, Lisa Wirtz, Iliana Tantcheva-Poor, Wolfram Malter, Max Zinser, Thomas Bieber, Dagmar Knebel-Mörsdorf

**Affiliations:** a Center for Biochemistry, University Hospital Cologne, University of Cologne, Cologne, Germany; b Department of Pediatrics, University Hospital Cologne, University of Cologne, Cologne, Germany; c Department Cell Biology of the Skin, University Hospital Cologne, University of Cologne, Cologne, Germany; d Cologne Excellence Cluster on Cellular Stress Response in Aging-Associated Diseases, University Hospital Cologne, University of Cologne, Cologne, Germany; e Department of Dermatology, University Hospital Cologne, University of Cologne, Cologne, Germany; f Department of Gynecology and Obstetrics, University Hospital Cologne, University of Cologne, Cologne, Germany; g Department of Plastic, Reconstructive and Aesthetic Surgery, University Hospital Cologne, University of Cologne, Cologne, Germany; h Department of Dermatology and Allergy, Christine Kühne-Center for Allergy Research and Education, University Hospital Bonn, Bonn, Germany; University of Arizona

**Keywords:** HSV-1, atopic dermatitis, human skin, viral entry, epidermal barriers, IL-4/IL-13

## Abstract

To infect its human host, herpes simplex virus 1 (HSV-1) must overcome the protective barriers of skin and mucosa. Here, we addressed whether pathological skin conditions can facilitate viral entry via the skin surface and used *ex vivo* infection studies to explore viral invasion in atopic dermatitis (AD) skin characterized by disturbed barrier functions. Our focus was on the visualization of the onset of infection in single cells to determine the primary entry portals in the epidermis. After *ex vivo* infection of lesional AD skin, we observed infected cells in suprabasal layers indicating successful invasion in the epidermis via the skin surface which was never detected in control skin where only sample edges allowed viral access. The redistribution of filaggrin, loricrin, and tight-junction components in the lesional skin samples suggested multiple defective mechanical barriers. To dissect the parameters that contribute to HSV-1 invasion, we induced an AD-like phenotype by adding the Th2 cytokines interleukin 4 (IL-4) and IL-13 to healthy human skin samples. Strikingly, we detected infected cells in the epidermis, implying that the IL-4/IL-13-driven inflammation is sufficient to induce modifications allowing HSV-1 to penetrate the skin surface. In summary, not only did lesional AD skin facilitate HSV-1 penetration but IL-4/IL-13 responses alone allowed virus invasion. Our results suggest that the defective epidermal barriers of AD skin and the inflammation-induced altered barriers in healthy skin can make receptors accessible for HSV-1.

**IMPORTANCE** Herpes simplex virus 1 (HSV-1) can target skin to establish primary infection in the epithelium. While the human skin provides effective barriers against viral invasion under healthy conditions, a prominent example of successful invasion is the disseminated HSV-1 infection in the skin of atopic dermatitis (AD) patients. AD is characterized by impaired epidermal barrier functions, chronic inflammation, and dysbiosis of skin microbiota. We addressed the initial invasion process of HSV-1 in atopic dermatitis skin to understand whether the physical barrier functions are sufficiently disturbed to allow the virus to invade skin and reach its receptors on skin cells. Our results demonstrate that HSV-1 can indeed penetrate and initiate infection in atopic dermatitis skin. Since treatment of skin with IL-4 and IL-13 already resulted in successful invasion, we assume that inflammation-induced barrier defects play an important role for the facilitated access of HSV-1 to its target cells.

## INTRODUCTION

After invasion via mucosal surfaces or skin, herpes simplex virus 1 (HSV-1) establishes primary infection in the epithelium of its human host and becomes latent in neuronal ganglia. Reactivation can lead to recurrent viral shedding near the site of initial infection. HSV-1 generally causes mild infections; however, immune deficiencies and dysregulation are implicated in some severe infections (1). Host and viral factors that are associated with the outcome of HSV infections are mostly not well understood. Preconditions for successful viral invasion in tissue are presumably epithelial breaks due to mechanical injuries. Alternatively, disturbed epithelial integrity and loss of barrier function under pathological conditions such as inflammatory responses or preexisting infections might facilitate viral invasion. Patients with atopic dermatitis (AD) can be seriously affected by disseminated HSV skin infections (termed eczema herpeticum) which, at least in children, usually results from a primary HSV-1 infection (2). Since AD represents a chronic inflammatory skin disease with a complex pathophysiology (3, 4), the risk factors for higher susceptibility to HSV-1 include multiple parameters ranging from impaired epidermal barriers and dysbiosis of skin microbiota to dysregulated immune responses which are most likely mutually reinforcing processes (5–8). In this study, we addressed whether it is a precondition for eczema herpeticum that the lesional skin of AD patients facilitates the initial steps of HSV-1 invasion.

Successful initiation of viral infection in tissue requires the access of viral glycoproteins to its host receptors. Cellular entry of HSV-1 includes attachment to heparan sulfate proteoglycans on the cell surface, followed by the interaction of the viral glycoprotein D (gD) with its receptors, which in turn initiates the fusion of the viral envelope with cellular membranes (9, 10). The primary gD receptors for HSV-1 on human cells are the cell-cell adhesion protein nectin-1 and herpesvirus entry mediator (HVEM), a member of the tumor necrosis factor receptor superfamily (11, 12). Thus far, less is known about how HSV-1 gains access to its receptors in the epithelium to initiate infection.

To dissect the relevance of physical epidermal barriers for HSV-1 invasion, we established an *ex vivo* infection model of murine and human skin (13–15). Once the dermis is separated, murine and human epidermal sheets are highly susceptible to HSV-1 via the basal layer upon *ex vivo* infection; however, the virus cannot penetrate full-thickness skin via the apical surface confirming the effective outside-in barrier function of the skin (14, 15). Even in the absence of the cornified layer, a functional tight-junction (TJ) barrier effectively inhibits infection of lower layers in murine skin (16). Remarkably, mechanical wounds of the human skin surface do not provide *ad hoc* entry portals for HSV-1 upon *ex vivo* infection (15). Here, we explored the role of pathological skin conditions for successful penetration of HSV-1.

AD pathogenesis involves multidirectional interactions of immune dysregulation, microbial dysbiosis and barrier dysfunction (4, 17). Physical barrier dysfunctions are based on alterations of multiple components of the stratum corneum and the TJs which rely on genetic defects and/or inflammation-induced modifications. The best-known dysfunctions comprise the altered lipid and protein structures of the stratum corneum with filaggrin mutations as genetic predisposition for AD (18, 19) and increased risk for eczema herpeticum (20). In addition, TJ proteins are implicated in AD, such as decreased claudin-1 expression, which correlated with disease severity and impaired barriers that may enhance susceptibility to HSV-1 infection (21–24).

In this study, we *ex vivo* infected lesional skin samples to investigate whether AD skin *per se* allows HSV-1 invasion via the skin surface. Since the virus indeed successfully penetrated lesional skin, we visualized key players in barrier function which supported multiple impairments of the physical barriers. To dissect the complex cross talk of barrier function and immune responses that lead to conditions facilitating HSV-1 invasion, we investigated whether the induced Th2 immune response in skin without preexisting barrier defects is sufficient for HSV-1 infection. Intriguingly, after *ex vivo* infection of interleukin-4 (IL-4)/IL-13-treated skin, we observed infected cells supporting that Th2 responses already enabled the virus to overcome the protective skin barrier and gain access to its receptors to initiate infection.

## RESULTS

### *Ex vivo* infection of AD skin.

**FIG 1:**
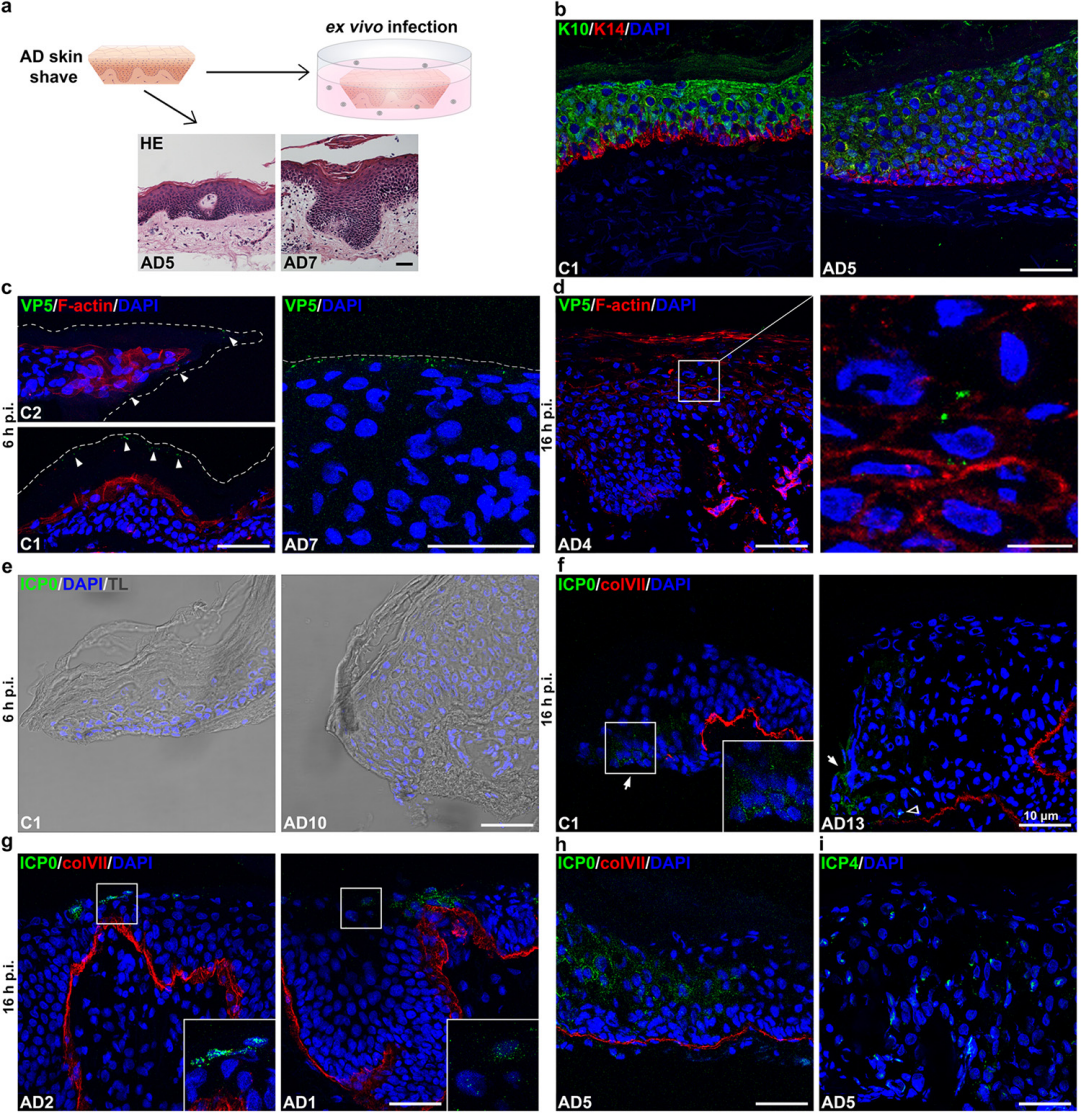
HSV-1 penetration in AD skin shaves. (a) Schematic illustrating *ex vivo* infection of AD skin shaves. HE-stained sections visualize samples with characteristics of commencing (AD5) and acute eczema (AD7). (b to i) After infection with HSV-1 at a multiplicity of infection (MOI) of ca. 100 PFU/cell, cross sections of control (C) and AD skin shaves with DAPI (blue) as nuclear counterstain are shown. Collagen VII (colVII; red) depicts the basement membrane. Scale bars, 50 μm. (b) K10/K14 immunostainings indicate differentiation defects in the eczematic AD skin, including expanded suprabasal layers and nuclei in the stratum corneum. (c) VP5-positive virus particles (green) (arrowheads) at (C2) or below (C1) the control skin surface at 6 h p.i. F-actin (red) depicts the cell morphology. AD skin with virus particles in the stratum corneum containing cell nuclei. Dashed lines indicate sample borders. (d) VP5-positive virus particles (green) underneath the granular layer as shown in the magnification at 16 h p.i. (e) Transmission light (TL) visualizes sample edges with no ICP0-expressing cells at 6 h p.i. (f) At 16 h p.i., nuclear (open arrowhead) and cytoplasmic (arrows) ICP0-expressing cells (green) at the sample edges are shown. (g) ICP0-expressing cells (green) in the granular layer and (h) throughout the epidermis. (i) Nuclear ICP4-expressing cells (green) throughout the epidermis.

To explore whether AD predisposes the epidermis to HSV-1 invasion, we *ex vivo* infected lesional skin samples from AD patients and compared them to control skin from healthy donors. Shave biopsy specimens comprising the epidermis and a thin dermal layer were taken from AD patients with severe disease (SCORAD ≥ 30) (Table 1) and from healthy donors. Histological analyses determined the state of eczema for each sample which ranged from commencing, subacute, acute to chronic eczema (Fig. 1a and Table 1). All AD samples showed the characteristic epidermal thickening (Fig. 1b), which results from the altered epidermal growth and keratinocyte terminal differentiation (25). To confirm the impaired differentiation programs of the epidermis, we performed K10/K14 stainings (see Fig. 5, below). Lesional skin was characterized by expanded basal keratin 14 (K14) and suprabasal keratin 10 (K10) positive layers leading to enlargement of the epidermis (Fig. 1b).

**TABLE 1:** Characteristics of AD samples

Sample	Age (yrs)	SCORAD	IgE concn (IU/mL)	Eczema^*a*^	Location of infection^*b*^	
					16 h p.i.	24 h p.i.
AD1	41	61	6,307	NA	**Apical cells**	NA
AD2	27	66	19.7	NA	**Apical cells**	NA
AD4	35	NA	47.7	chr	**Apical cells**	**All layers**
AD5	55	65	55.6	com	**All layers**	**All layers**
AD6	55	63	8,322	com	Only edges	Only edges
AD7	47	39	799	acu	Only edges	Only edges
AD8	32	55	2,175	sub	**All layers**	Only edges
AD10	56	65	85	acu	Only edges	**All layers**
AD11	38	47	5,850	acu	Only edges	NA
AD12	20	80	13,552	acu	Only edges	Only edges
AD13	58	66	14,236	chr	Only edges	NA

a Eczema (chr, chronic; com, commencing; acu, acute; sub, subacute) categorized at time of sample taking.

b Boldfacing indicates infection via the skin surface.

The heterogeneity of the lesional skin samples allowed us to investigate HSV-1 penetration in AD epidermis under variable conditions (Table 1). After *ex vivo* infection of skin shaves with HSV-1 at ca. 100 PFU/cell, we initially visualized the presence of virus particles by staining the capsid protein VP5. Viruses were visible at the sample edges of control skin shaves, where some viruses accumulated on or in the most upper part of the cornified layer at 6 h postinfection (p.i.) (Fig. 1c). In AD skin samples, we observed virus particles in the cornified layer at this early time, while few particles were found underneath the granular layer at 16 h p.i. suggesting viral penetration (Fig. 1c and d). The granular layer represents the uppermost nucleated epidermal layer under healthy conditions while nucleated cells can be also present in the cornified layer of eczematic skin (see Fig. 5). To explore in more detail whether viruses successfully entered individual cells in AD epidermis via the external skin surface, we visualized the very early expressed viral protein ICP0. Once HSV-1 penetrates cellular membranes and the viral genome is released into the nucleus, ICP0 first localizes in the nucleus and then relocalizes to the cytoplasm during later infection, indicating viral replication (13, 26). After submerging control (*n* = 3) or AD skin shaves (*n* = 7) in virus suspension for 6 h, no ICP0-expressing cells were detected even at the sample edges (Fig. 1e). At 16 h p.i., both control (*n* = 3) and AD skin shaves (*n* = 11) showed infected keratinocytes at sample edges (Fig. 1f), where loss of tissue integrity was visualized by histological analyses (data not shown). In addition, disruption of the basement membrane at the edges was shown by discontinuous staining of its component collagen VII (Fig. 1f). In line with previous results of various skin samples from healthy donors (15), infected cells were limited to the sample edges in control skin. However, in some AD skin samples (*n* = 3) we detected single or patches of infected cells in the granular layer with nuclear ICP0, indicating the completion of successful viral entry or cytoplasmic ICP0, which demonstrated the onset of viral replication (Fig. 1g). Furthermore, AD skin samples (*n* = 2) with areas of infected cells throughout the epidermal layers were found at 16 h p.i. (Fig. 1h), while another sample (*n* = 1) showed infected areas only at 24 h p.i. (Table 1). These infected areas exhibited a continuous collagen VII staining reflecting an intact basement membrane and showed no infection in the underlying dermal layer. Thus, HSV-1 most likely gained access to the viable epidermis by passing the cornified layer and the TJs (Fig. 1h). To confirm the ICP0 staining pattern throughout the epidermis, we additionally visualized the viral protein ICP4 which marks the nuclear deposition of viral DNA (27). In line with ICP0, the nuclear ICP4 stainings depicted areas with infected cells throughout all epidermal layers (Fig. 1i). Taken together, the *ex vivo* infection studies revealed that AD skin allowed HSV-1 to penetrate via the epidermal surface. The extent of infected cells did not correlate with the state of eczema, SCORAD, or IgE levels (Table 1). Thus, we conclude that a variety of AD conditions can facilitate the initial step of infection.

**FIG 2:**
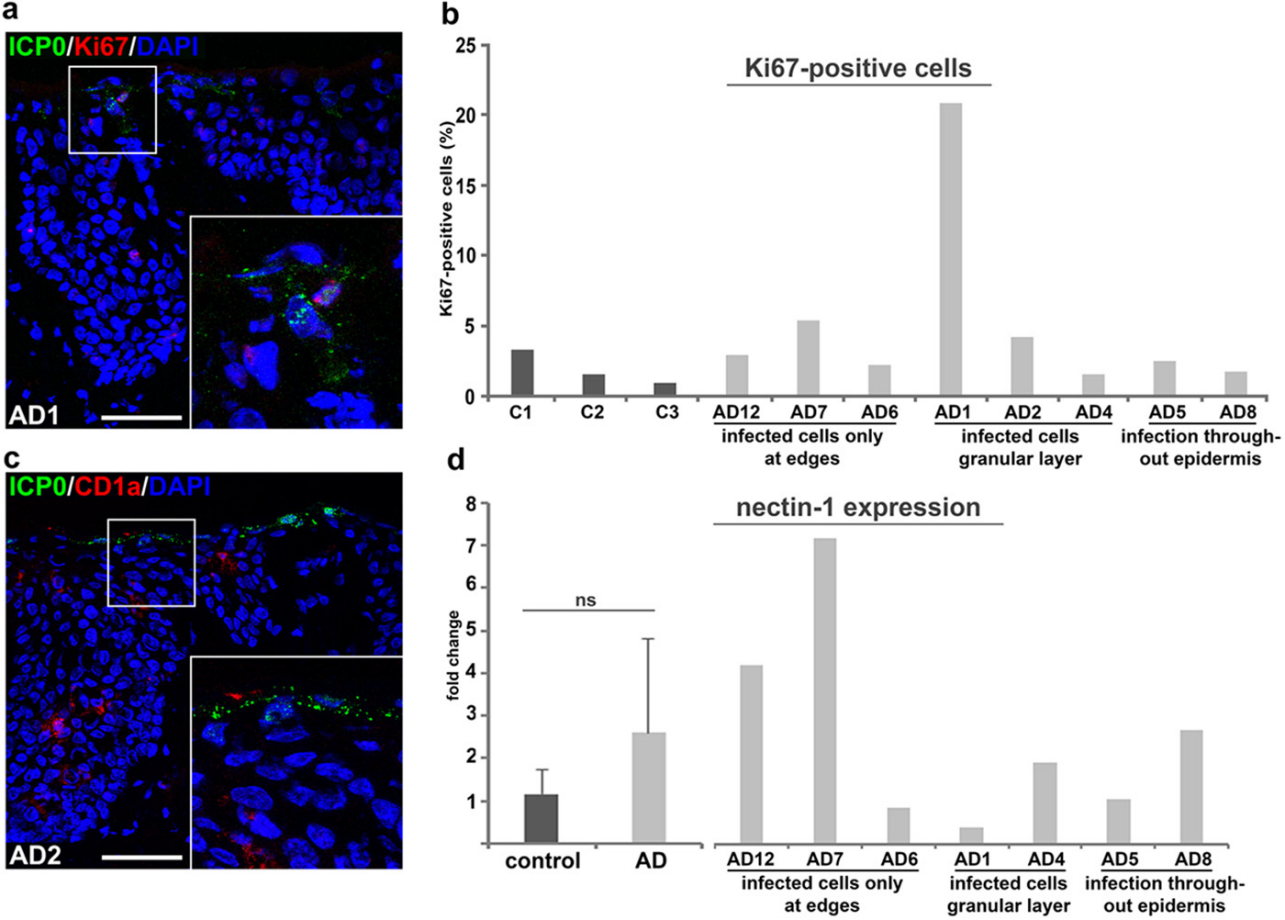
Characterization of HSV-1 penetration in AD skin shaves. (a) Costaining of Ki67 and ICP0 indicates very few proliferating cells with infection. (b) Quantification of Ki67-positive cells is shown for each sample and demonstrates variable numbers in AD (*n* = 8) compared to control (*n* = 3) skin shaves. (c) Costaining of CD1a (red) and ICP0 (green) shows no infected Langerhans cells. (d) qRT-PCR demonstrates variable nectin-1 transcript levels in AD (*n* = 7) compared to control (*n* = 3) skin shaves. Nectin-1 expression is shown for each infected AD sample.

Since AD is a hyperproliferative skin disease, we performed costainings of the marker Ki67 and ICP0 which demonstrated that proliferative keratinocytes were not preferentially infected (Fig. 2a). In addition, no enhanced infection was found in highly proliferative samples compared to samples with fewer Ki67-positive cells (Fig. 2b).

We next explored whether HSV-1 invades AD skin via epidermal Langerhans cells (LCs). Next to the barrier function of the cornified layer (stratum corneum), TJs, restricted to the granular layer, form a further protective barrier (see Fig. 5, below). The elongated dendrites of LCs can penetrate TJs without disturbing their barrier function which, in turn, might allow pathogen uptake (28, 29). In general, HSV-1 can infect epidermal LCs in human skin (30). Costaining of the LC-specific surface antigen CD1a (31) and ICP0 revealed no infected Langerhans cells in areas of infected keratinocytes (Fig. 2c). Thus, we suggest that extended dendrites of Langerhans cells do not provide a preferred entry portal for HSV-1 invasion but that keratinocytes represent the initial targets upon *ex vivo* infection of the AD skin samples.

To address whether the susceptibility of lesional AD skin to HSV-1 correlated with enhanced receptor presence, we analyzed nectin-1 expression. At least in murine epidermis, HSV-1 entry strongly depends on nectin-1, while HVEM acts as alternative receptor (32). In human epidermis, approximately 40 to 85% of the analyzed epidermal cells express nectin-1 on their surfaces (15). Here, we determined nectin-1 expression by qRT-PCR which demonstrated a high variation among the AD skin samples but no significant difference to control skin (Fig. 2d). Interestingly, the high nectin-1 transcription levels in some AD skin samples did not correlate with enhanced viral invasion (Fig. 2d). These results are a first hint that receptor accessibility rather than receptor presence allows viral entry; however, localization studies of nectin-1 are needed to demonstrate where nectin-1 is present.

### Redistributed barrier components in the AD skin shaves.

**FIG 3:**
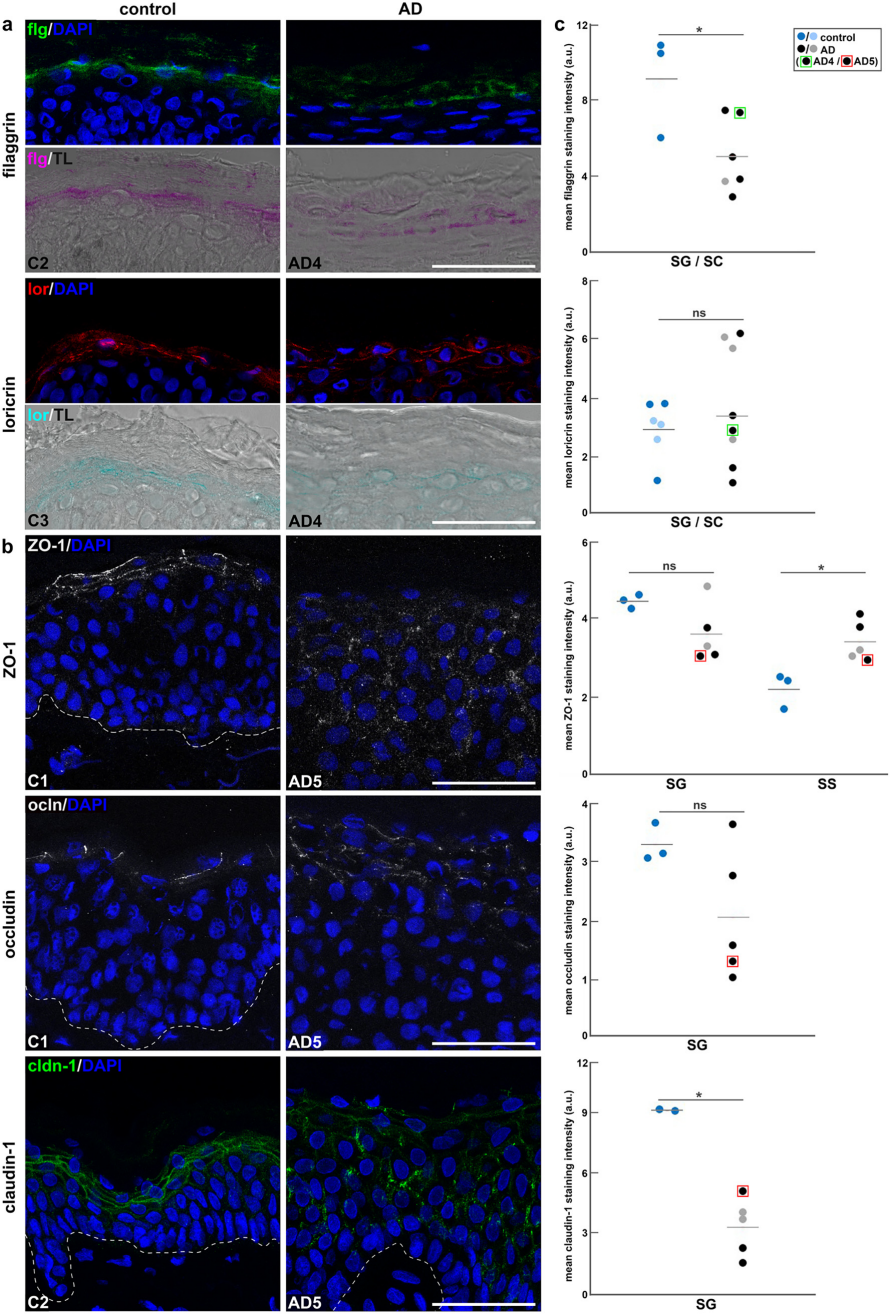
Redistribution of barrier components in AD skin. (a) Immunostainings of skin shaves show redistributed filaggrin (flg) and loricrin (lor) in AD compared to control (C) skin with DAPI (blue) as nuclear counterstain. Transmission light (TL) visualizes the morphology of the stratum corneum (SC) and the stratum granulosum (SG). (b) Immunostainings of AD skin shaves show redistribution of ZO-1, occludin (ocln), and claudin-1 (cldn-1) in the suprabasal layers compared to control shaves. Dashed lines represent the basement membrane. Scale bars, 50 μm. (c) Quantification of fluorescence shows intensities in control skin shaves (dark blue) (*n* = 3) and breast skin (light blue) (*n* = 3) versus AD skin shaves with detectable (black) (*n* = 3 to 5) or undetectable apical infection (gray) (*n* = 1 to 3). In AD skin shaves, filaggrin staining intensities are significantly lower in the SG in the stratum granulosum and the corneum (SG/SC). The redistribution of ZO-1 to the stratum spinosum led to significantly higher intensities the stratum spinosum (SS) led to significantly compared to control shaves. The discontinuous claudin-1 stainings in AD skin shaves showed significantly lower intensities in the stratum granulosum (SG) compared to control shaves. *P* value (*) ≤ 0.05.

Our *ex vivo* infection studies of AD skin shaves imply that HSV-1 can overcome both barriers, the stratum corneum and the TJs, to reach its receptor nectin-1, which, as a component of adherens junctions, is located underneath TJs (33). Whether nectin-1 is also expressed on apical surfaces of granular keratinocytes has not yet been determined. Next, we explored the physical barrier modifications in the AD skin samples to address their potential contribution to successful infection. Because of its multifunctional role in barrier formation, we visualized filaggrin in the AD shaves. As expected, filaggrin stainings were less intense in AD (*n* = 6) compared to control (*n* = 3) skin samples, suggesting reduced filaggrin expression and alterations of the cornified envelope (Fig. 3a and c). Furthermore, we stained the differentiation marker loricrin, another barrier component of the squamous layer that is downregulated in AD skin (34). Staining intensities of loricrin were quite heterogenous in AD skin, suggesting a variable extent of impaired differentiation (Fig. 3a). Control skin samples (*n* = 3) showed reduced heterogeneity of loricrin stainings, which was confirmed by additional analyses of breast skin (*n* = 3) from healthy donors (Fig. 3c). Interestingly, reduced filaggrin or loricrin stainings correlated with viral invasion in most AD samples (Fig. 3c).

We then stained TJ components, including the scaffolding protein ZO-1 and the integral membrane proteins occludin and claudin-1. The distinct distribution of ZO-1 in the granular layer, as shown in the control skin (*n* = 3), was replaced by a punctate staining pattern in AD shaves (*n* = 7), while all AD samples (*n* = 11) showed additional ZO-1 stainings underneath the granular layer in the spinous layer supporting an impaired TJ barrier (Fig. 3b). Occludin, which was visible at the apical cells of the granular layer in control skin (*n* = 3), was redistributed in AD shaves (*n* = 11), as visualized by the punctate pattern throughout the suprabasal layers with some heterogeneity in staining intensities (Fig. 3b and c). Finally, we analyzed claudin-1 distribution. As observed for ZO-1 and occludin, claudin-1 stainings were quite distinct in the granular layer of control skin (*n* = 3), while they were reduced in the granular and redistributed to the spinous layer in AD skin (*n* = 11) (Fig. 3b). The significantly lower staining intensities in AD shaves suggest decreased claudin-1 expression (Fig. 3c), which agrees with previous observations (35).

Taken together, all staining patterns confirmed dysregulated barrier components in AD skin. While all AD samples showed reduced claudin-1 stainings, the patterns of ZO-1 and occludin stainings were quite heterogenous among the samples (Fig. 3c), which, in turn, might influence the extent of dysfunctional barriers. Since AD samples with reduced ZO-1 or occludin in addition to low claudin-1 levels were infected from the skin surface (Fig. 3c, black circles), we assume that AD conditions associated with severely impaired TJ barrier promote HSV-1 invasion. This assumption is in line with previous findings in murine skin where functional TJs interfere with HSV-1 entry (16).

### *Ex vivo* infection of IL-4/IL-13-treated human skin.

**FIG 4:**
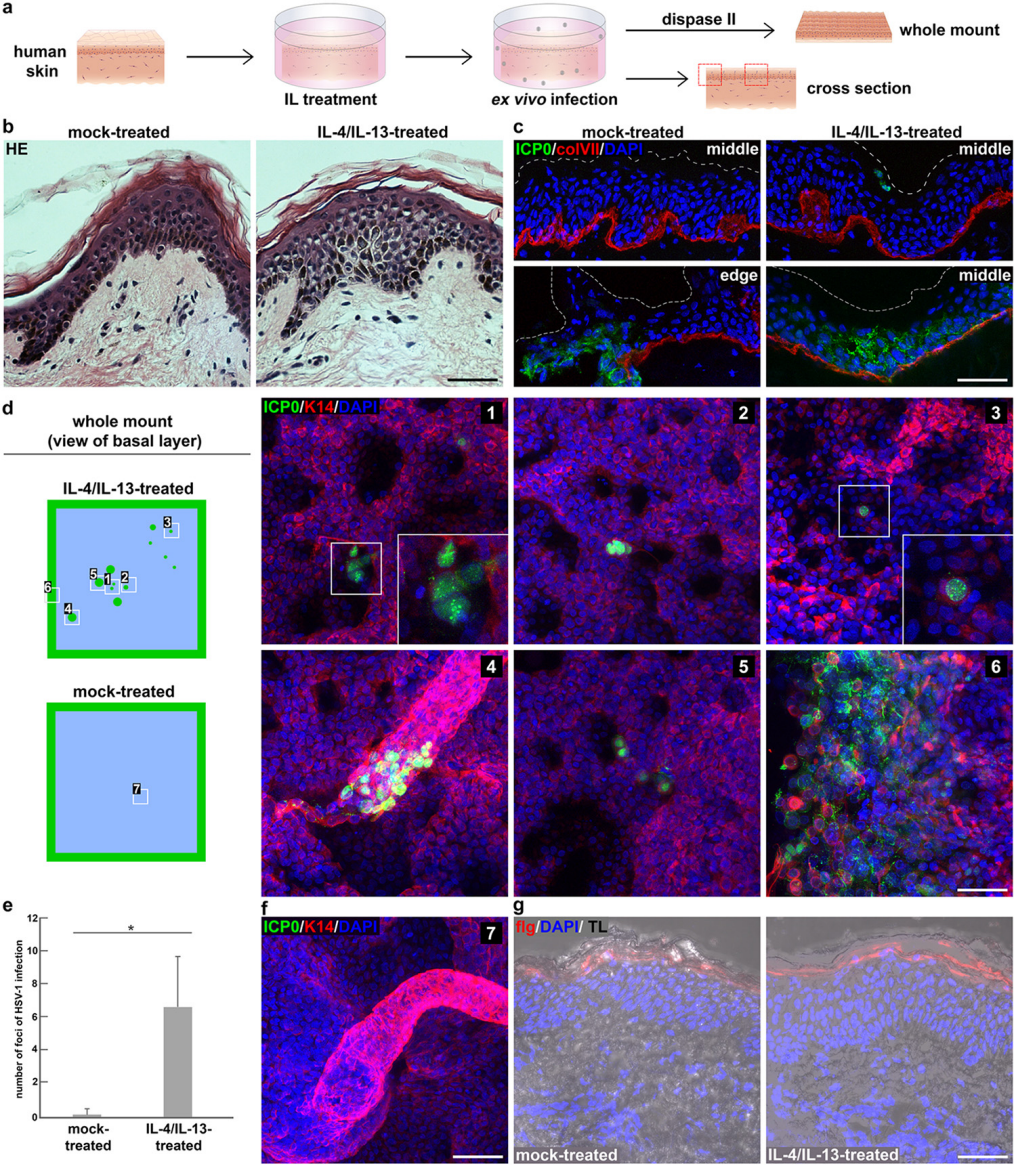
HSV-1 entry in IL-4/IL-13-treated human skin. (a) Schematic illustrating IL-4/IL-13-treatment of full-thickness human skin (*n* = 5) followed by *ex vivo* infection and analyses of infected cells. Epidermal whole mounts were prepared after infection by dispase II treatment to show the distribution of the infected cells in the basal layer. Localization of the immunostainings at the edge or middle of the cross sections is indicated with dotted boxes (red). (b) HE staining visualizes discrete spongiosis after IL-4/IL-13-treatment of abdominal skin sample for 3 days. (c) After infection of skin with HSV-1 at ca. 100 PFU/cell for 24 h, cross sections show ICP0-expressing cells (green) only at edges after mock-treatment. Single infected (green) suprabasal cells and infected cell layers are visible after IL-4/IL-13-treatment. DAPI (blue) serves as a nuclear counterstain, and collagen VII (colVII; red) depicts the basement membrane. Dashed lines represent the apical sample border. (d) Scheme of epidermal whole mount prepared 24 h p.i. showing the distribution of infected cells. Immunostainings of epidermal whole mounts from IL-4/IL-13-treated skin visualize ICP0-expressing single cells (green) (2, 3) and cell clusters at the hair follicles (4), in the interfollicular epidermis (1, 5), and at the sample edge (6). K14 stainings depict basal keratinocytes. (e) Quantification of infected areas in mock-treated (seven replicates from three individuals) and IL-4/IL-13-treated human skin (ten replicates from four individuals). *, *P* ≤ 0.05. (f) Uninfected hair follicle of mock-treated skin 24 h p.i. (g) Cross sections show comparable filaggrin (red) distribution in mock- and IL-4/IL-13-treated skin. Transmission light (TL) visualizes the skin morphology. Scale bars, 50 μm.

Since Th2 cytokine-driven inflammation and induction of impaired barriers play key roles in AD (36), the Th2 cytokines IL-4 and IL-13 were employed in various human skin models to mimic an AD-like-Th2-driven inflammatory response (37–39). We dissected the parameters that contribute to HSV-1 invasion in AD skin by treating human skin from healthy donors with IL-4/IL-13 to explore whether IL-induced modifications of the epidermal phenotype can *per se* facilitate viral penetration. After treatment of full-thickness skin samples prepared from breast (*n* = 3) and abdominal skin (*n* = 2) with IL-4 and IL-13 for 3 days, we observed marked intercellular edema (discrete spongiosis) (Fig. 4a and b), which represents a hallmark of AD. After infection of IL-4/IL-13-treated skin with HSV-1 at ca. 100 PFU/cell for 24 h, we indeed found single ICP0-expressing cells underneath the cornified layer, though rather rarely in cross sections (Fig. 4c), while the sample edges were well infected. In addition, a patch of infected cells in suprabasal layers away from the edges was occasionally detected (Fig. 4c). In mock-treated skin (*n* = 5), ICP0-expressing cells were exclusively detected at sample edges (Fig. 4c). To better visualize the rare events of infected cells in IL-4/IL-13-stimulated skin, we infected full-thickness breast (*n* = 2) and abdominal skin (*n* = 2) and then separated the dermis from the epidermis to prepare epidermal whole mounts (Fig. 4a). Intriguingly, the view on the basal side of all epidermal samples revealed multiple spots of infected cells ranging from mostly single cells to small cell clusters (Fig. 4d). Quantification illustrates 3 to 12 infected spots per whole-mount preparation after IL-4/IL-13-treatment, while 1 to 2 spots were detected in few mock-treated samples, though only very close to the edges (Fig. 4e). Intriguingly, some of the hair follicles in IL-4/IL-13-treated skin showed infected cells close to the interfollicular epidermis, which we never observed at hair follicles from mock-treated skin (Fig. 4d and f). These results suggest that modifications in IL-4/IL-13-stimulated skin may lead to viral access via the hair shaft. In murine skin, we found that cells in the bulge and hair germ of hair follicles are in principle susceptible to HSV-1, but only after *ex vivo* infection of epidermal sheets with preserved hair follicles (14).

Our initial attempt to visualize potential barrier alterations included filaggrin since it is known as a critical barrier component in AD skin. After IL-4/IL-13-treatment, we observed no obvious redistribution or decreased intensities of filaggrin stainings (Fig. 4g). The results, taken together, show that *ex vivo* infection with HSV-1 revealed successful viral penetration in IL-4/IL-13-stimulated skin, supporting that the virus can gain access via the external skin surface due to the Th2 cytokine-induced modifications.

## DISCUSSION

Only a subset (~3%) of AD patients develops eczema herpeticum, suggesting that in addition to gaining access to its receptors in AD skin, HSV-1 must also find conditions of various dysregulated immune responses that allow a widespread infection. Thus far, very little is known about the predisposing factors that are associated with the severe outcome of HSV-1 infection (8, 40). The assumption that impaired barrier function correlates with facilitated viral penetration was recently supported by a three-dimensional skin barrier dysfunction model that enables HSV-1 infection (41). Moreover, clinical trials showed that treatment with the monoclonal antibody dupilumab, which inhibits IL-4/IL-13 signaling, was associated with a decreased risk of eczema herpeticum. Thus, improved AD severity is thought to be related to a decrease of eczema herpeticum; however, the underlying mechanism still remains elusive (42). We investigated whether the disturbed barrier function of AD skin and/or the induction of Th2 inflammatory responses *per se* is sufficient to allow HSV-1 to initiate infection in the epidermis.

**FIG 5:**
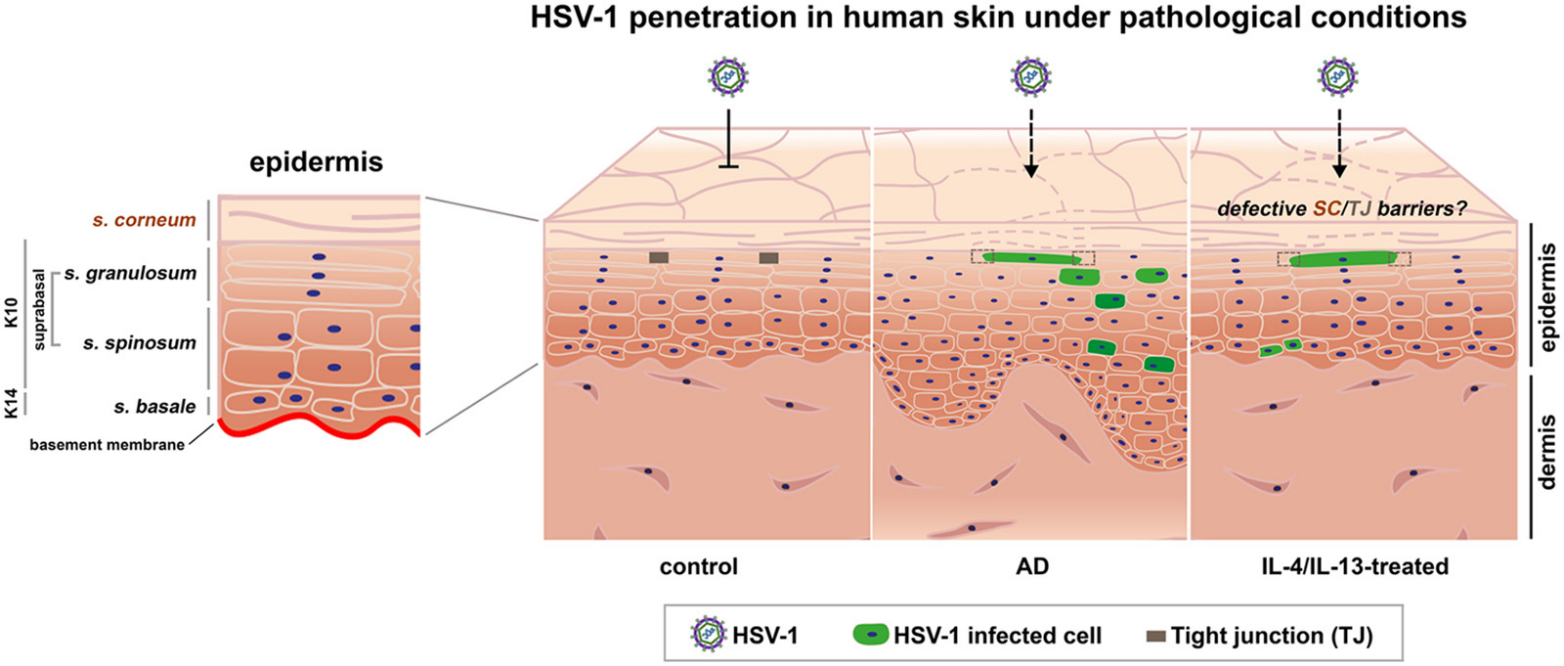
Schematic representation of how HSV-1 penetrates human skin under pathological conditions. Schematic illustrating the structure of human epidermal layers with respective markers. The stratum corneum (SC) and the tight junctions (TJs) form protective barriers to inhibit virus invasion via the skin surfaces of healthy individuals. Atopic dermatitis (AD) skin characterized by epidermal thickening, disturbed SC barriers (dotted lines), and impaired TJs (dotted boxes) offers the virus access to keratinocytes throughout the epidermis to initiate infection. After IL-4/IL-13-treatment of human skin, infected cells are detected which might result from an impaired stratum corneum (SC) and dysfunctional TJs.

Our *ex vivo* infection studies in AD skin samples revealed infected cells in suprabasal layers away from the edges. Since we never observed infection of the epidermis by HSV-1 passing through the dermal layer in healthy (15) or AD skin shaves, we conclude that the virus invades the lesional AD skin surface. The precondition for successful invasion is that the impaired barrier function allows the virus to overcome both the stratum corneum and the TJ barriers. Visualization of filaggrin and loricrin, as well as ZO-1, occludin, and claudin-1, demonstrated that all barrier components were redistributed in the AD compared to the control skin samples although to various extents in each sample. The number of AD samples and the complexity of pathological modifications made it difficult to identify a distinct pattern of redistributed barrier components that could be directly attributed to facilitated invasion of HSV-1. Impaired barrier function in AD is mostly determined by measuring transepidermal water loss implying the impairment of the TJ barrier (4). In our experiments, HSV-1 served as a sensor to support that the barrier functions, including the stratum corneum and the TJs, are disrupted to an extent that allows viral penetration in AD lesions (Fig. 5). Under *in vivo* conditions, this initial step of infection might be facilitated by the enhanced Staphylococcus aureus colonization of AD skin (43) since these microbes can target TJs, leading to disturbed junctional integrity (44). The development of a disseminated HSV-1 infection in a subset of AD patients additionally relies on the extent of immune dysregulations.

As soon as 3 h p.i., HSV-1 infects basal keratinocytes if the virus can directly access receptors in the basal layer of epidermal sheets separated from the dermis (15). ICP0-expressing cells in AD skin were not detected before 16 to 24 h p.i., suggesting that the receptors were not easily accessible throughout the epidermis. Expression of the receptor nectin-1 was comparable in AD and control skin samples; however, we cannot exclude that impaired differentiation in AD skin leads to a redistribution of nectin-1 that may facilitate access for the virus.

The finding that AD skin samples could be infected with HSV-1 irrespective of the eczema state suggests that successful invasion depends on the structural outcome of dysregulated immune responses rather than on transient inflammatory microenvironments. Future experimentation will address whether facilitated viral invasion relies on barrier defects of genetic origin by analyzing infection of nonlesional AD skin. Here, we stimulated healthy skin with IL-4/IL-13 prior to infection to explore the impact of the Th2 cytokine responses on HSV-1 invasion. Various mouse models helped to establish the role of IL-4/IL-13 in AD pathogenesis and revealed their contribution to allergic inflammation and attenuated barrier function often based on the downregulation of antimicrobial peptides and filaggrin (45). Supplementation of human skin equivalents with IL-4/IL-13 supports that the Th2 inflammation supports hyperproliferation, impaired lipid composition, and reduced skin protein expression, influencing keratinocyte integrity and barrier function (38, 46).

In IL-4/IL-13-stimulated human skin, we observed penetration of HSV-1 via the skin surface, although to a limited and variable extent (Fig. 5). Clusters of single infected cells with either nuclear or cytoplasmic ICP0 expression at 24 h p.i. indicated that some cells are still in the early phase of infection, while others already initiated viral replication. We assume that the IL-4/IL-13 stimulation induced initial alterations in the skin samples that sufficiently alter the integrity of cell-cell junctions that allow the virus to reach its receptors and initiate infection (Fig. 5). Although some viruses evolved strategies to alter cell-cell junctions (47), HSV-1 is thought to opportunistically rely on conditions that lead to junctional disruption that facilitates the accessibility of receptors. How receptor accessibility might be achieved after supplementation with IL-4/IL-13 could rely on various conditions. Recent studies of IL-4/IL-13-treated stratified human keratinocytes showed increased HSV-1 expression which may correlate with the induction of reduced filaggrin expression. This observation led to the speculation that filaggrin deficiency modifies the local pH and thereby enhances viral entry (48). Whether and how IL-4/IL-13 treatment of skin induces impaired physical barriers that allow viral invasion or whether further induced modifications influence receptor distribution and successful invasion is open for future investigation.

## MATERIALS AND METHODS

### Preparation of human skin.

Skin shaves of lesional skin from various areas were taken with a scalpel from patients (*n* = 11) with diagnosed AD (Table 1). The estimated state of eczema is based on hematoxylin and eosin (HE) stainings (Table 1). Immediately after surgery, skin samples were transported in Dulbecco modified Eagle medium (DMEM)/high-glucose/GlutaMAX (Life Technologies) with 10% fetal calf serum (FCS), penicillin (100 IU/mL), and streptomycin (100 μg/mL) and prepared for infection. Control skin shaves were taken from the upper leg of healthy donors (*n* = 3) comparably to AD patients. In addition, shave biopsy specimens (*n* = 3 individuals) were prepared from skin derived from patients undergoing breast surgery as described previously (15, 49). All skin shaves had a comparable size (ca. 3 × 3 mm). For IL treatment, full-thickness skin samples, which were taken from patients undergoing breast (*n* = 3 individuals) or plastic surgery (*n* = 2 individuals), were cut in pieces (ca. 4 × 4 mm) after removal of subcutaneous fat (49). The size of breast and abdominal skin samples allowed infection experiments with two to three replicates per explant. After infection, epidermal whole mounts from total skin were prepared by using dispase II (4 U/mL; Roche) treatment overnight at 4°C to separate the epidermis from the dermis (49).

### Ethics statement.

Human skin specimens were obtained after informed consent from all patients. The study was approved by the Ethics Commission of the Medical Faculty, University of Cologne (approval 17-481).

### Interleukin treatment.

Immediately after surgery, total human skin samples were treated with IL-4 (25 ng/mL) and IL-13 (25 ng/mL) diluted in DMEM/high-glucose/GlutaMAX (Life Technologies) with 10% FCS, penicillin (100 IU/mL), streptomycin (100 μg/mL), and 0.05% bovine serum albumin (BSA). After 3 days, mock- and IL-4/IL-13-treated samples were infected with HSV-1 at ca. 100 PFU/cell for 24 h in the absence of IL-4/IL-13.

### Virus.

Infection studies were performed with HSV-1 wild-type (Glasgow) strain 17+ from purified virus preparations obtained from the supernatant of infected BHK cells, as described previously (49). The calculation of the virus dose was based on the estimated cell number (ca. 2.5 × 10^5^) of the superficial areas of skin shaves and full-thickness skin. HSV-1 was given to the tissue samples at 37°C defining time point zero. Skin shaves and total skin were infected at ca. 100 PFU/cell by submerging them in virus-containing medium for various times (49).

### Histochemistry, immunocytochemistry, and antibodies.

For HE staining, shaves and full-thickness skin samples were fixed with 3.4% formaldehyde overnight at 4°C or for 2 h at room temperature. Fixed samples were prepared as paraffin sections (8 μm), as described previously (49). The morphology of all samples was assessed by HE stainings.

For cryosections, skin samples were embedded in OCT compound (Sakura), frozen at −80°C, and cut into 8 μm thick cross sections (49). Cryosections were fixed with 2% formaldehyde for 10 min at room temperature, and epidermal whole-mount preparations were fixed with 3.4% formaldehyde overnight at 4°C (49). For stainings of claudin-1, occludin, and ZO-1, cryosections were fixed with ice-cold ethanol for 30 min and then with acetone (–20°C) for 3 min. Sections of total skin and shaves were incubated with primary antibodies overnight at 4°C, followed by incubation with the species-specific Alexa Fluor-conjugated secondary antibodies and DAPI (4′,6′-diamidino-2-phenylindole) for 45 min at room temperature. Epidermal whole mounts were incubated with primary antibodies overnight at room temperature and with the secondary antibodies and DAPI overnight at 4°C. The following primary antibodies were used: mouse anti-ICP0 (MAb 11060; 1:60) (50), mouse anti-ICP4 (MAb 58S; 1:1,000), mouse anti-VP5 (MAb DM165; 1:1,000) (51), mouse anti-collagen VII (1:500; Santa Cruz Biotechnology), rabbit anti-loricrin (1:1,000; BioLegend), mouse anti-CD1a (1:50) (31), mouse anti-claudin-1 (1:500; A9; Santa Cruz), mouse anti-filaggrin (1:500) (AKH1; Santa Cruz), mouse anti-occludin (1:400; OC-3F10; Thermo Fisher Scientific), rabbit anti-ZO-1 (1:400; Thermo Fisher Scientific), rabbit anti-K10 (1:1,000; BioLegend), guinea pig anti-K14 (1:150; GP-CK14; Progen), and rabbit anti-Ki67 (1:400; Thermo Fisher Scientific). F-actin was labeled with phalloidin-Atto 565 (1:2,000; Sigma) for 45 min at room temperature.

Microscopy of several continuous sections per sample was performed using an epifluorescence microscope (Zeiss Axiophot) equipped with a Nikon Digital Sight camera system (DS-2MV)/NIS Elements software (for HE stainings) and a Leica DM IRBE microscope linked to a Leica TCS-SP/5 confocal unit. Images were assembled using Photoshop (Elements 2018; Adobe) and Illustrator (version CS5; Adobe). Confocal projections and merged images are shown. Images were analyzed using Fiji (version 2.0.0-rc-65/1.51s) (52) by measuring the mean fluorescence intensity of three different areas per sample.

### RNA preparation and qRT-PCR.

RNA was isolated from 150 μm thick cryosections of skin shaves embedded 6 h p.i. by using an RNeasy Plus minikit (Qiagen). cDNA was synthesized using SuperScript II reverse transcriptase (Life Technologies). qPCRs were performed using the SYBR GreenER qPCR SuperMix Universal (Life Technologies) on the DNAengine-Opticon 2 System (Bio-Rad). Nectin-1-specific primers (forward, 5′-CTGCAAAGCTGATGCTAACC-3′; reverse, 5′-GATGGGTCCCTTGAAGAAGA-3′) were used; for normalization, RPLP0 (60S acidic ribosomal protein P0) primers (forward, 5′-ACTCTGCATTCTCGCTTCCT-3′; reverse, 5′-GGACTCGTTTGTACCCGTTG-3′) were used. The efficiency for each primer pair was determined, and the relative expression levels were calculated using the threshold cycle (ΔΔ*C_T_*) method.

### Statistics.

For statistical analyses, Student *t* tests were performed to calculate *P* values using the unpaired two-tailed method. Differences were considered to be statistically significant with *P* values of ≤0.05 (*).
